# Pesticides: Cysteine Assistance

**DOI:** 10.1289/ehp.115-a78b

**Published:** 2007-02

**Authors:** Carol Potera

The organophosphate and carbamate pesticides used today act by blocking acetyl-cholinesterase (AChE), an enzyme needed for proper functioning of the nervous system in insects, humans, and other animals. These pesticides, which contaminate air, water, soil, and food, are toxic not only to insects, but also to people and other animals. Now Yuan-Ping Pang, director of the Mayo Clinic’s Computer-Aided Molecular Design Laboratory, has discovered an insect-specific region on AChE that could presage a new generation of better-targeted pesticides.

Current pesticides bind the amino acid serine at the active site of AChE. In work described in the 1 January 2007 issue of *Bioorganic & Medicinal Chemistry Letters*, Pang created three-dimensional computer models based on genomic information of AChEs obtained for the greenbug (*Schizaphis graminum*) and the English grain aphid (*Sitobion avenae*), which decimate wheat, barley, and sorghum worldwide. Models of the active site revealed that the amino acid cysteine occurs at a particular location (dubbed C289) in the two insects, but not in people. “We inspected the entire active site of the human enzyme and couldn’t find one cysteine residue,” says Pang. A sequence analysis of AChEs from 68 species obtained from GenBank also detected C289 in cockroaches, lancelets, rice beetles, bollworms, silkworms, honey bees, moths, and armyworms.

Because C289 is located at the entrance to the active site of AChE, it potentially could react with chemical pesticides designed to target that enzyme. Some early experiments in Pang’s laboratory have shown that the lone cysteine can snag reactive chemicals and damage the enzyme. “For the first time, we have a blueprint to make a new generation of pesticides that are toxic only to pests,” Pang notes.

Next Pang created a computer model of AChEs in the malaria-carrying *Anopheles* mosquito. In addition to the insect-specific C289 detected in the first study, he identified an insect-specific arginine, R339, at another location of the active site. The discovery of these two targets makes it possible to “create an effective new pesticide that specifically kills mosquitoes,” says Pang, “and potentially revolutionize the way we control mosquito-caused diseases.” The discovery of other such combinations could yield any number of new pesticides that target particular pests while sparing beneficial species such as honey bees. The mosquito study appears in the December 2006 inaugural issue of the online journal *PLoS ONE*.

AChE is one of the most important targets for the chemical control of many agricultural and medical insect pests, according to entomologist Kun Yan Zhu at Kansas State University, whose laboratory first sequenced the cDNA that encodes AChE in the greenbug. The discovery of insect-specific AChE regions “could potentially lead to the development of novel pesticides that would be expected to be toxic to insects but nontoxic or less toxic to humans,” Zhu says.

## Figures and Tables

**Figure:**
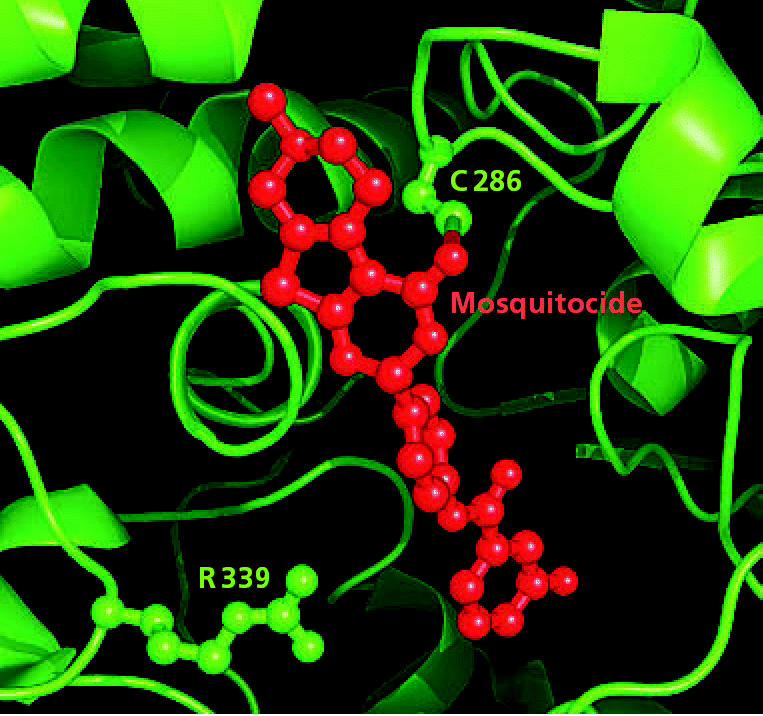
Bug-specific breakthrough A model shows two active sites (C289 and R339) where mosquito-specific pesticides may bind with the AChE enzyme. These two sites are found only in insects and thus may lead to pesticides that are safer for humans.

